# Reviewed article: Gonçalves HAO, Martorell LB, Freire MCM. Surveillance of public water supply fluoridation from the perspective of municipal health department technicians: a cross-sectional study, Goiás, 2022-2023. Epidemiol Serv Saude. 2026:35;e20250444

**DOI:** 10.1590/S2237-96222026v35e20250444.a

**Published:** 2026-03-16

**Authors:** Sandra Maria do Valle Leone de Oliveira

**Affiliations:** 1 Fundação Oswaldo Cruz, Campo Grande, MS, Brazil Fundação Oswaldo Cruz Campo Grande MS Brazil

## First round comments

O trabalho aborda tema relevante para a saúde pública, porém necessita revisão substancial para alinhar resumo, discussão e conclusão ao objetivo proposto e aos dados coletados.

há necessidade de revisão linguística e editorial, com foco em:Correções gramaticais e ortográficas pontuais; Padronização terminológica e normativa;Sugestões para maior clareza e concisão em trechos redundantes ou pouco objetivos; Adequações de estilo científico (evitar juízos subjetivos ou informais como “chama a atenção”).

Deixo alguns exemplos para o autor considerar e REVISAR todo o texto:

a. “...foi utilizado um questionário eletrônico autoaplicável, contendo perguntas fechadas e algumas abertas...” sugiro um ajuste para melhorar fluidez: “Utilizou-se questionário eletrônico autoaplicável, com perguntas fechadas e abertas…” b. “...convocada pelos gestores da Superintendência de Vigilância em Saúde da Secretaria de Estado da Saúde.”c. eliminar repetições: “...convocada pela Superintendência de Vigilância em Saúde estadual.” d. “Os resultados acerca da vigilância da fluoretação da água encontram-se na Tabela 2.” sugiro “A Tabela 2 apresenta os resultados sobre a vigilância da fluoretação da água.”“Chama a atenção o relato de um dos respondentes que considerou o fluoreto como veneno…”Recomenda-se evitar juízo subjetivo sugiro “Destaca-se o relato isolado de um técnico que classificou o fluoreto como veneno…”e.“Há, portanto, necessidade de adequações na estratégia…” Tornar mais científico: “Evidencia-se, portanto, a necessidade de aprimorar a estratégia…” “...até mesmo os trabalhadores do SUS.” talvez “...inclusive entre profissionais do Sistema Único de Saúde (SUS).” “...remetendo à importância de incentivos financeiros...” talvez “...ressaltando a importância da alocação de recursos financeiros…”

Por favor, revise todo o texto para ajustar a ortografia e gramática da lingua portuguesa.

Entre os pontos técnico-científicos:

1- A conclusão do resumo é uma breve descrição geral da importância da fluoretação, mas não respondem diretamente se as ações de vigilância aos objetivos propostos e se a fluoretação apartir da perspectiva dos entrevistados são adequadas, insuficientes ou heterogêneas. Reformular para refletir achados: nível de implementação, lacunas de capacitação, influência de estrutura laboratorial e apoio estadual, recomendações práticas.

2- Resumo: incluir método, n de municípios/técnicos, 2-3 resultados chave e conclusão alinhada ao objetivo e na conclusão responder explicitamente se a vigilância é adequada, identificar principais gargalos e propor recomendações factíveis.

3- Introdução: Trazer uma visão geral dessa estratégia globalmente. Há países que adotam como o Japão, Europa e há outros países que optaram por outra metodologia de fluoretação.

3-Discussão: Atenção aos pontos descritos abaixo:

Relacionar resultados com literatura e normas brasileiras (VIGIÁGUA, Portaria GM/MS 888/2021):Explorar os determinantes estruturais e operacionais mencionados pelos técnicos.

Inserir as limitações como por exemplo o viés de opinião, a falta de dados laboratoriais secundários.

Nas Tabelas/Figuras: apresentar distribuição dos municípios segundo frequência de monitoramento, resultado dos testes de flúor e barreiras relatadas.

4- Considerar no método:

Desenho: Caso seja transversal com questionário a técnicos, explicitar critérios de seleção e taxa de resposta (para avaliar viés).Utilizar um check list para guiar a descrição metodológica, como por exemplo o STROBE ou o que considerar mais apropriado.

Análise: Se houve abordagem quali-quantitativa, descrever método de análise temática e medidas descritivas (média de flúor, proporção de municípios monitorados).

5- Acesso ao banco de dados: a informação deve ser corrigida seguindo a politica editorial da revista.

“Os dados de pesquisa (como bancos de dados, códigos, métodos e outros materiais utilizados ou resultantes da pesquisa) devem, preferencialmente, ser depositados em repositórios de dados confiáveis, como o SciELO Data (lista disponível em https://wp.scielo.org/wp-content/uploads/Lista-de-Repositorios-Recomendados_pt.pdf), com um identificador persistente, como o DOI (Digital Object Identifier).

O acesso a esses dados, por meio de um link para o repositório, deve ser informado na seção “Disponibilidade de dados” e a referência completa deve ser incluída na seção de “Referências”, conforme exemplo abaixo:

Andrade M. Estudo de genes em ratos albinos na América Latina. OSF [dataset], 2018, ASM0000v1. http://dx.doi.org/10.1590/0123-45620187214”

Instruções: “Disponibilidade dos dados do artigo: declaração sobre o acesso aos dados de pesquisa (bancos de dados, códigos, métodos e outros materiais utilizados e resultantes da pesquisa), informar link do

repositório e referenciamento, com a devida citação no texto”

Recommendation: Major revision

## Second round comments

A maior parte das recomendações foi implementada de forma satisfatória; a versão R1 traz dados inéditos que qualificam a análise situacional e respaldam as conclusões.

Permanecem alguns tópicos que podem ser melhorados ou necessitam ser melhor esclarecidos:

1-Quanto ao tipo de estudo: estudo é transversal, descritivo‑analítico, baseado em questionário aplicado a técnicos municipais. Para evitar expectativas de ensaio de efetividade, sugiro marcar a modalidade como “Estudo descritivo transversal”.

2-Validação e confiabilidade do instrumento: Inclua um breve relato sobre os passos de construção do questionário (revisão de literatura, validação aparente por especialistas, pré‑teste) e, se possível, um índice de consistência interna (p. ex. Alfa de Cronbach para blocos temáticos). Caso não seja possível, inclua a ausência dessa etapa como uma limitação do estudo. Caso a coleta já esteja finalizada, mencione um plano para aferir a confiabilidade em futuras etapas ou estudos derivados.

3-Torne o parágrafo conclusivo mais objetivo, incorporando números‑chave:“Apenas 15 % dos municípios realizam monitoramento mensal e 8 % dispõem de laboratório próprio; mais de 60 % dos técnicos relatam carência de capacitação.”Isso reforça a conexão entre achados e recomendações práticas.

3.1 Indicadores de heterogeneidade: Considere acrescentar uma figura simples (mapa de calor ou gráfico de barras) mostrando a distribuição da frequência de análise do fluoreto por município ou por região administrativa. Facilita a visualização de áreas prioritárias.

4-Fortalecer discussão operacional : Ao comentar barreiras, vincule‑as a recomendações concretas (p. ex. “Implantar contrato de cooperação intermunicipal para uso compartilhado de laboratório de referência”, “Incluir módulo sobre fluoretação em cursos regulares da VIGIÁGUA”). Não se esqueça de que ao recomendar opções para enfrentar o problema, deve utilizar evidências cientiíficas.

4.1 Compare seus achados com experiências exitosas de outros estados ou países, mesmo que em poucas linhas, para contextualizar a factibilidade das propostas.

5-Taxa de resposta e possíveis vieses, por exemplo :mantenha o cálculo de 100 % (194/194), mas mencione explicitamente a exclusão posterior de 25 questionários e a possível introdução de viés de informação (ex.: questionários parcialmente incompletos, má interpretação de itens).

6-Revisão final de linguagem e estilo: Apesar da melhoria, revise frases longas que possam ser divididas para maior clareza.Prefira verbos na voz ativa e evite expressões vagas como “é necessário”, substituindo por “recomenda‑se”. Mesmo para recomendação utilize evidências científicas.

6-Atualize as referências ou justifique o uso. Se o limite permitir (30 referências), inclua pelo menos um estudo avaliativo nacional ou internacional sobre vigilância da fluoretação para ampliar a discussão comparada.

Se o limite permitir (30 referências), inclua pelo menos um estudo avaliativo nacional ou internacional sobre vigilância da fluoretação para ampliar a discussão comparada.

Resumo estruturado

Garanta que cada seção (Introdução, Métodos, Resultados, Conclusão) não ultrapasse 2-3 frases e que contenha: delineamento, n final (169 técnicos de X municípios), 2‑3 números principais e recomendação resumida. 

Recommendation: Major revision

## Third round comments

Prezado autor, são necessários ajustes menores.

1-Revisão de linguagem e estilo - Parcialmente atendido.

Texto está mais fluido, porém persistem pontuais correções:“eraconcursada” → “era concursada”. “precupante” → “preocupante”: Duplicação “às as”.

Recomendo leitura final de prova para padronizar “fluoreto/fluoretação”, hífens e percentuais.

2- Inconsistência numérica sobre SISAGUA.

Resultados/Tabela 4 indicam 65,4% dos municípios que monitoravam inseriam dados no SISAGUA, mas a Discussão cita “apenas 41,1%”. Harmonizar (provável valor correto: 65,4%).

2-Ajustar as pequenas falhas tipográficas/gramaticais listadas; revisar duplicações e padronizar termos/abreviações (SISAGUA, VIGIAGUA).

3-Padronização de denominadores nas Tabelas 4 e 5.

4-Reafirmar no texto, onde pertinente, se os percentuais usam N=26 (apenas municípios que analisam) ou o total da amostra; isso evita leituras ambíguas.

Recommendation: Minor revision

